# Drivers of Carbon Export Efficiency in the Global Ocean

**DOI:** 10.1029/2018GB006158

**Published:** 2019-07-22

**Authors:** Stephanie Henson, Fred Le Moigne, Sarah Giering

**Affiliations:** ^1^ National Oceanography Center European Way Southampton UK; ^2^ GEOMAR Helmholtz Center for Ocean Research Kiel Kiel Germany; ^3^ Now at Mediterranean Institute of Oceanography, UM 110, Aix Marseille Univ., Université de Toulon, CNRS, IRD Marseille France

**Keywords:** export flux, export efficiency, MAREDAT, remineralization

## Abstract

The export of organic carbon from the surface ocean forms the basis of the biological carbon pump, an important planetary carbon flux. Typically, only a small fraction of primary productivity (PP) is exported (quantified as the export efficiency: export/PP). Here we assemble a global data synthesis to reveal that very high export efficiency occasionally occurs. These events drive an apparent inverse relationship between PP and export efficiency, which is opposite to that typically used in empirical or mechanistic models. At the global scale, we find that low PP, high export efficiency regimes tend to occur when macrozooplankton and bacterial abundance are low. This implies that a decoupling between PP and upper ocean remineralization processes can result in a large fraction of PP being exported, likely as intact cells or phytoplankton‐based aggregates. As the proportion of PP being exported declines, macrozooplankton and bacterial abundances rise. High export efficiency, high PP regimes also occur infrequently, possibly associated with nonbiologically mediated export of particles. A similar analysis at a biome scale reveals that the factors affecting export efficiency may be different at regional and global scales. Our results imply that the whole ecosystem structure, rather than just the phytoplankton community, is important in setting export efficiency. Further, the existence of low PP, high export efficiency regimes imply that biogeochemical models that parameterize export efficiency as increasing with PP may underestimate export flux during decoupled periods, such as at the start of the spring bloom.

## Key Points

- An apparent inverse relationship between PP and export efficiency is found globally, driven by rare very high export efficiency
- Zooplankton and bacteria data suggest (de)coupling between PP and upper ocean remineralization processes drives transient high export efficiency
- A biome scale analysis shows that the factors affecting export efficiency may be different at regional and global scales

## 1 Introduction

The ocean's biological carbon pump is a major factor in determining the air‐sea partitioning of CO_2_ (Kwon et al., 2009); without it, atmospheric CO_2_ concentration would be ~200 ppm higher than currently (Parekh et al., 2006). The pump starts with the uptake of CO_2_ by phytoplankton during primary production (PP) and subsequent sinking of organic carbon from the upper ocean. Only a fraction of PP leaves the upper ocean, known as the export efficiency (export flux/PP). The export efficiency is affected by multiple factors such as phytoplankton community structure (which may affect formation of aggregates or sinking by ballasting; Buesseler, 1998; Boyd & Newton, 1999; Francois et al., 2002), zooplankton (which package phytoplankton into fast‐sinking fecal pellets or may transfer carbon directly to depth via diel vertical migration; Cavan et al., 2015; Dagg et al., 2014; Steinberg et al., 2002), and bacterial remineralization (Belcher et al., 2016; Buchan et al., 2014; Buesseler et al., 2007; Le Moigne et al., 2016).

Each of these factors varies seasonally and regionally, and therefore, the export efficiency is unlikely to be constant either spatially or over the course of a year, as has been demonstrated in both observational (Buesseler, 1998; Henson et al., 2012) and modelling (Henson et al., 2015; Wassmann, 1998) studies. Previous studies have posited the concept of tight coupling between primary producers and their grazers which is only broken intermittently, either during the onset of the spring bloom or by transient forcing such as an eddy, allowing a strong export pulse to occur (e.g., Brix et al., 2006; Rii et al., 2008; Wassmann, 1998). Model results suggest that at high latitudes in spring, PP increases rapidly, outpacing the growth of the grazing population so that organic carbon is retained in the upper ocean, resulting in low export efficiency (Henson et al., 2015). In summer, the same study suggests that grazing is able to keep pace with PP so that the flux of organic carbon via fecal pellets increases, resulting in high export efficiency. An alternative scenario, developed based on observations, is that early in the growing season, in the absence of grazers, PP sinks out rapidly in the form of aggregates (Briggs et al., 2011; Buesseler, 1998; Lam et al., 2011), resulting in high export efficiency. As the bloom progresses, zooplankton grazing and other upper ocean remineralization processes increase, establishing recycling pathways and leading to low export efficiency (Wassmann, 1998). In both scenarios, the degree of coupling (or decoupling) between PP and the processes affecting how much organic carbon is exported results in temporally variable export efficiency. However, whether seasonal decoupling of PP and export results in low or high export efficiency remains unclear.

Here we investigate the potential mechanisms driving periods of extremes in export efficiency by combining large data sets of export flux, satellite‐derived estimates of PP and phytoplankton community structure, and global databases of bacteria, mesozooplankton, and macrozooplankton abundance.

## 2 Methods

Export flux measurements made using the ^234^Th technique were taken from the data synthesis of Le Moigne et al. (2013) and updated to include an additional 140 observations published from 2014 to 2016 (Haskell et al., 2013; Le Moigne et al., 2015, 2016; Owens et al., 2015Planchon et al., 2015; Puigcorbé et al., 2015; Rosengard et al., 2015) for a total of 821 data points. All data report export fluxes from a depth of 100 m (standard deviation ±28 m). Buesseler and Boyd (2009) suggest calculating export flux from the base of the euphotic zone, however the necessary ^234^Th profile data needed to recalculate the literature values is rarely available. Normalizing the reported ^234^Th data to the euphotic depth would require an assumption regarding the form and rate of flux attenuation (e.g., an assumption that flux attenuation conforms to a Martin curve and assumption of a “b” value; Martin et al., 1987). We therefore do not attempt to recalculate the reported literature values of POC flux. In high latitudes or bloom periods, this may result in an underestimate of export flux and vice versa in low latitude or oligotrophic regions (although not necessarily; e.g., Jacquet et al., 2011; Puigcorbé et al., 2015; Rosengard et al., 2015).

To calculate the export efficiency, we take into account the residence time of ^234^Th, which is ~2–20 days (Coale & Bruland, 1985). We therefore use satellite‐derived PP integrated over the 16 days prior to the observation to calculate the export efficiency (i.e., the *ThE*_*i*_‐ratio; sensu Henson et al., 2011). To calculate satellite‐derived PP, climatological, 8‐day composites of Level 3 SeaWiFS chlorophyll concentration, photosynthetically available radiation and AVHRR SST were downloaded from https://oceancolor.gsfc.nasa.gov/and https://podaac.jpl.nasa.gov/AVHRR-Pathfinder, respectively. The Carr (2002) algorithm for PP was applied and all data averaged onto a 1° grid. The Carr (2002) PP data, when applied in an empirical algorithm, were previously found to replicate export measurements well (Henson et al., 2011). The spatial averaging mitigates possible effects of (sub)mesoscale variability and lateral advection on export flux in the absence of information on either. Our results and conclusions are not sensitive to using alternative satellite‐derived PP algorithms (Behrenfeld & Falkowski, 1997; Westberry et al., 2008; see Figures S1–S4 in the supporting information).

Estimates of the contribution to PP of different size fractions of phytoplankton (micro, pico, or nano) were obtained from satellite‐derived data (Uitz et al., 2010). The estimates are a monthly climatology (1998–2007) at 1° resolution and represent PP over the “productive layer,” which is defined as 1.5 times the euphotic depth. For each export efficiency estimate, the corresponding seasonal average, size‐fractionated PP was identified. Seasons were defined in 3‐month periods, for example, December–March for Northern Hemisphere winter. Export efficiency estimates were aligned with seasonally averaged data to ensure sufficient data coverage in ecosystem variables (see below). A total of 670 coincident export and phytoplankton size data were available.

Observations of mesozooplankton biomass, macrozooplankton abundance (200 μm–2 mm and >2 mm, respectively), and bacterial abundance were taken from the MARine Environment DATa (MAREDAT) databases (Buitenhuis et al., 2012; Moriarty et al., 2013; Moriarty & O'Brien, 2013). The data were provided as monthly climatologies with 1° resolution and at 33 vertical levels. Only data from the top 100 m were used in this analysis to correspond to the export measurements. For zooplankton, sufficient data existed to evaluate the seasonal mean abundances coincident with export efficiency estimates (*n* = 149 for mesozooplankton and *n* = 59 for macrozooplankton). However, bacterial abundance data had to be averaged annually to generate sufficient overlap with the export efficiency estimates (*n* = 223). For each export efficiency estimate, corresponding observations were identified within a 1° spatial range for zooplankton data or within 3° for bacteria data (again, due to a paucity of bacterial abundance data). The possible use of bacterial production data was also investigated; however, data density was too poor to allow sufficient overlap with the export flux data set (Figure S5).

Monthly climatological 1° resolution mixed layer depth, and nitrate and silicate concentration data were obtained from de Boyer Montegut et al. (2004) and the World Ocean Atlas 2009 (Garcia et al., 2010), respectively. The mixed layer‐integrated nutrient concentrations are used to calculate the silica:nitrate drawdown ratio (ratio of maximum minus minimum mixed layer silica to maximum minus minimum mixed layer nitrate) and the nitrate drawdown corresponding to the month of export flux sampling (mixed layer nitrate at time of sampling as a fraction of maximum mixed layer nitrate). A total of 789 coincident export and nutrient data points were available.

**Figure 1:**
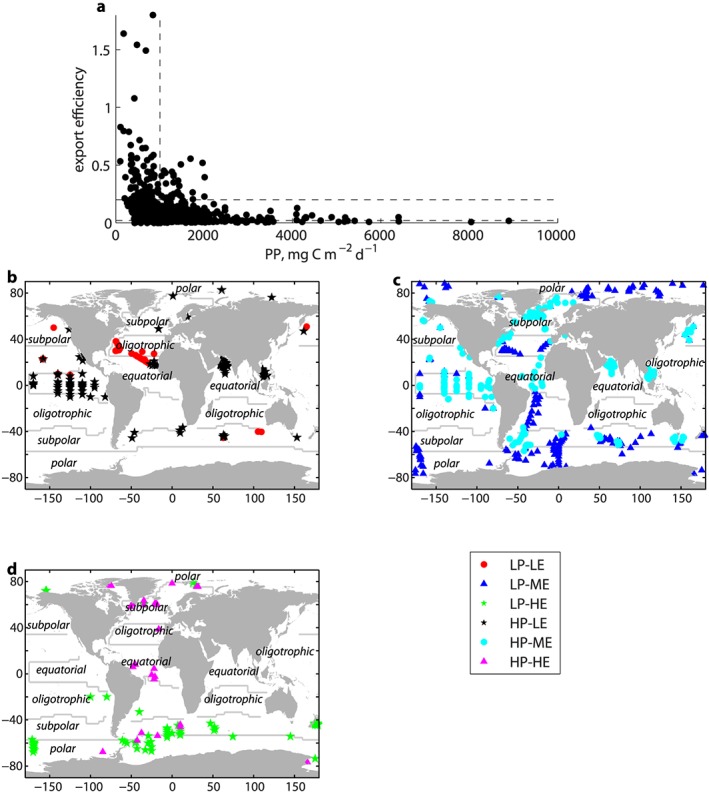
(a) Satellite‐derived primary productivity (PP) plotted against export efficiency (thorium‐derived export flux/PP). Horizontal dashed lines indicate threshold for high and low export efficiency regimes; vertical dashed line indicates threshold for high and low PP groups (see section 2 for details). Location of in situ samples marked as (b) low PP, low export efficiency, (LP‐LE) and low PP, high export efficiency (LP‐HE), (c) as low PP, moderate export efficiency (LP‐ME) low export efficiency and high PP, moderate export efficiency (HP‐ME), and (d) low PP, high export efficiency (LP‐HE) and high PP, high export efficiency (HP‐HE). See section 2 for definition of low/high regimes. Contours indicate location of biome boundaries (see Figure S6 for details).

To examine regional scale patterns in export efficiency and associated environmental conditions, we aggregated Longhurst provinces (Longhurst, 2007) into four broad biomes: equatorial, oligotrophic, subpolar, and polar. This ensured sufficient data within each province to undertake an analysis. Province boundaries are overlaid on Figure 1b and details of the aggregated Longhurst provinces are provided in Figure S6.

Differences in the mean of two groups were assessed with a one‐sided ANOVA test and deemed significant if they were different at the 95% level. Tables with the full results for all combinations of regimes and environmental factors is in Table S1 for global analysis and Table S2 for biome‐scale analysis. We draw the reader's attention to the small number of coincident export efficiency and ecosystem measurements in some regimes and for some variables (see individual figures). The results and interpretation of these cases necessarily carries an element of uncertainty.

## 3 Results

Primary productivity is plotted against export efficiency in Figure 1a. There is a clear suggestion of an inverse relationship between PP and export efficiency, as has previously been found for the Southern Ocean (Cavan et al., 2015; Laurenceau‐Cornec et al., 2015; Le Moigne et al., 2016; Maiti et al., 2013). However, our results suggest that even outside the Southern Ocean, export efficiency may be inversely proportional to PP. Note however that there is no relationship between export itself and PP (Figure S7), and the trend apparent in Figure 1a arises due to plotting PP against export/PP (i.e., reciprocal function). The apparent relationship is exaggerated by a relatively small number of data with low PP but high export efficiency Here we define high (low) PP conditions as greater (less) than 1,000 mg C m^−2^ d^−1^ (median of data set). Our analysis focusses on the extremes of export efficiency, and therefore, high export efficiency is defined as greater than 0.2 (eightieth percentile of data), low export efficiency is less than 0.02 (twentieth percentile), and moderate export efficiency lies between (Figure S8).

Almost all data points which exhibit low PP, high export efficiency characteristics (LP‐HE; *n* = 80) are located at high latitudes (Figure 1b). Low PP, low export efficiency (LP‐LE; *n* = 45) regimes are concentrated in the subtropical North Atlantic. High PP, low export efficiency (HP‐LE; *n* = 127) regimes are concentrated at low latitudes, principally in the equatorial Pacific and Arabian Sea, but can occasionally occur at high latitudes (Figure 1b). A very small number of data points (<4% of the data set) evince high productivity, high export conditions (HP‐HE; *n* = 29), which are confined to the Atlantic. Regimes with low PP and moderate export efficiency (LP‐ME; *n* = 277) not only occur mostly at higher latitudes in both hemispheres but also in parts of the subtropical Atlantic, whereas high PP, moderate export conditions (HP‐ME; *n* =263) are not frequently colocated with HP‐LE regimes in the equatorial Pacific and Arabian Sea but also occur in the North Atlantic and Argentine Basin.

The potential mechanisms underlying these regimes are explored by extracting information on the mixed layer nutrient conditions, phytoplankton size class, bacterial abundance, mesozooplankton biomass, and macrozooplankton abundance associated with each export efficiency estimate. In the following, we focus the discussion on the extremes of export efficiency (both low and high) in order to explore potential driving mechanisms; the conditions in moderate export efficiency regimes are also presented in the figures for completeness.

**Figure 2:**
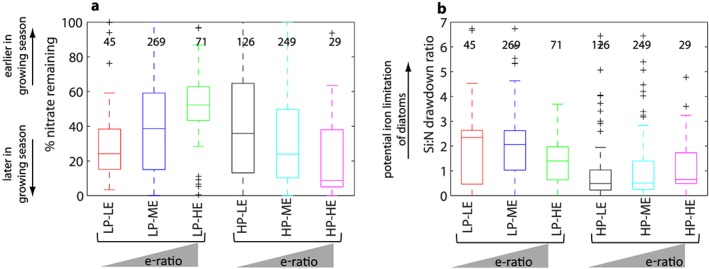
Box plots showing the mean and range in (a) percentage of maximum mixed layer nitrate remaining at time of export flux sampling and (b) mixed layer silicate:nitrate drawdown ratio for each of the export efficiency regimes identified in Figure 1b. The number of data points for each variable per regime is given in the plots. Statistical significance of the difference in means between regimes was tested using ANOVA; results are shown in Table S1.

Mixed layer‐integrated nitrate drawdown is used as a proxy for bloom phase at the time of sampling for export flux. Nitrate drawdown is significantly less at the time of sampling in LP‐HE than in all other regimes (Figure 2a), implying that LP‐HE regimes occur near the start of the phytoplankton growing season. The highest degree of nitrate drawdown is found for HP‐HE regimes suggesting that they occur toward the end of the phytoplankton growing season. Note however that we have insufficient seasonally resolved data to test this hypothesis. We also use the Si:N drawdown ratio to characterize ecosystem state. In the presence of sufficient nutrients and light, diatoms accumulate biomass with Si:N ~ 1 (Brzezinski, 1985); values in excess of 1 indicate over‐silicification of diatoms, which can occur due to iron limitation (Hutchins & Bruland, 1998). The Si:N ratio is typically >1 in low productivity conditions and <1 in high productivity conditions (Figure 2b).

**Figure 3:**
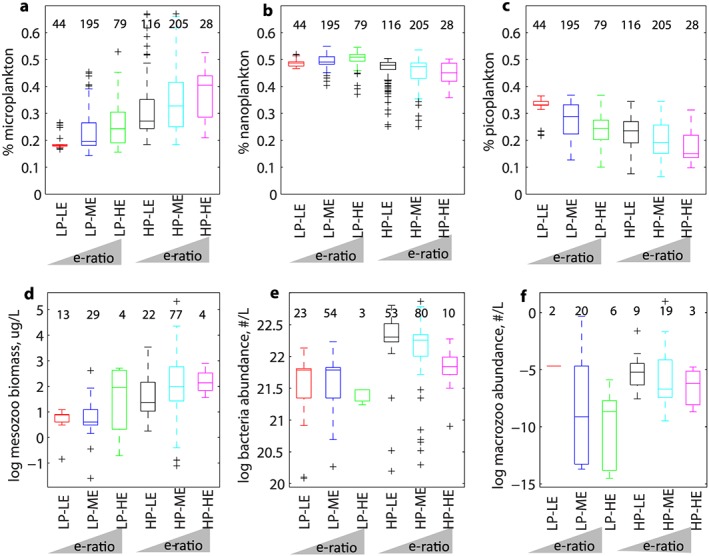
Box plots showing the mean and range in proportion of primary production assigned to 3 phytoplankton size classes—(a) micro, (b) nano, and (c) picoplankton—taken from satellite‐derived estimates (Uitz et al., 2010) and (d) mesozooplankton biomass, (e) bacterial abundance, and (f) macrozooplankton abundance (Buitenhuis et al., 2012; Moriarty et al., 2013; Moriarty & O'Brien, 2013) in each of the export efficiency regimes identified in Figure 1b. The number of data points for each variable per regime is given in the plots. Statistical significance of the difference in means between regimes was tested using ANOVA; results are shown in Table S1.

Figure 3 displays the ecosystem indicators associated with each of the export efficiency regimes. The data can be split into high and low PP groups, within each of which there are distinct trends in export efficiency associated with ecological conditions (Figure 3). For phytoplankton community structure (Figures 3a–3c), the high PP group is characterized by a higher proportion of microplankton and a lower proportion of picoplankton than the low PP group. Within these two groups, the trend is for the export efficiency to increase with increasing microplankton proportion but for the export efficiency to decrease with increasing picoplankton proportion. A similar pattern of two groups, separated by high/low PP, within which distinct trends with respect to export efficiency regime occur, is found for the zooplankton and bacteria data. For all three data sets, biomass or abundance is higher in high PP than low PP conditions. The trend is for increasing export efficiency with increasing mesozooplankton biomass, decreasing bacterial abundance, and decreasing macrozooplankton abundance. Box plots showing the mean conditions for the high PP and low PP groups can be found in Figure S9. In all cases, the means of the 2 groups are statistically different at the 95% level.

**Figure 4:**
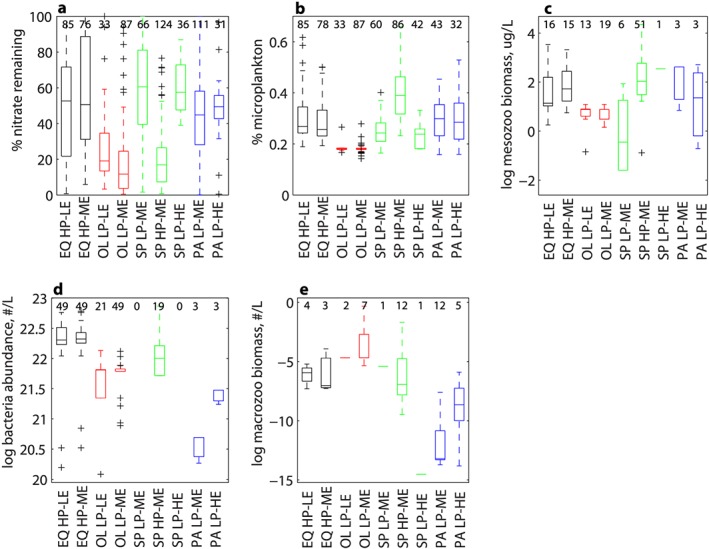
Selected box plots for dominant export efficiency regimes within each biome, showing the mean and range in (a) percentage of maximum mixed layer nitrate remaining at time of export flux sampling, b) proportion of primary production assigned to microplankton (Uitz et al., 2010), and (c) mesozooplankton biomass, (d) bacterial abundance, and (e) macrozooplankton abundance (Buitenhuis et al., 2012; Moriarty et al., 2013; Moriarty & O'Brien, 2013) in each of the export efficiency regimes identified in Figure 1b. Biome codes are as follows: EQ—equatorial, OL—oligotrophic, SP—subpolar, and PA—polar. Box plots of all environmental variables can be found in Figure S10. The number of data points for each variable per regime is given in the plots. Statistical significance of the difference in means between regimes was tested using ANOVA; results are shown in Table S2.

At the regional scale, each province tends to vary between two export states, generally while maintaining either high or low productivity. The number of data points in each biome and each export efficiency state can be found in Table S3. Selected plots of the environmental and ecological conditions associated with each biome and export efficiency state are in Figure 4, with full results in Figure S10. A table showing results of an ANOVA analysis are provided in Table S2. The equatorial biome is dominated by HP‐LE and HP‐ME conditions, with approximately equal frequency of each. In the oligotrophic biome, LP‐LE and LP‐ME conditions are prevalent, with LP‐ME occurring most frequently (Table S3). LP‐ME and HP‐ME conditions dominate the subpolar biome, with LP‐HE conditions also occurring less frequently. Finally, in the polar biome, LP‐ME conditions predominate with occasional LP‐HE states observed (Table S3). The majority of ANOVA tests shows that there is little difference in ecological or environmental conditions between the two export states of a particular biome (Table S2). The exception is the subpolar HP‐ME regime which has significantly lower proportion of nitrate remaining at time of sampling, higher microplankton abundance, and different mesozooplankton biomass than the subpolar LP‐ME or LP‐HE regimes. In the polar biome, LP‐HE conditions have significantly higher bacterial abundance and higher macrozooplankton abundance than LP‐ME regimes (Table S2). HP‐HE conditions do not dominate in any of the biomes (Table S3).

## 4 Discussion

The inverse relationship between PP and export efficiency previously identified for the Southern Ocean (Cavan et al., 2015; Laurenceau‐Cornec et al., 2015; Le Moigne et al., 2016; Maiti et al., 2013) is found to hold globally (Figure 1a). The apparent relationship is due to plotting a reciprocal function (i.e., PP vs. export/PP) and is exaggerated by a relatively small number of data with low PP and high export efficiency, that is, LP‐HE regimes. The inverse form is in direct contrast to canonical theory, which posits that as PP increases, a greater fraction of PP is exported. This latter is the functional form encapsulated in many global export efficiency algorithms (e.g., Laws et al., 2000; Laws et al., 2011) and implies that export flux increases at a faster rate than PP. The implicit conceptual model underlying these algorithms is that in highly productive regimes processes acting to remineralize PP in the upper ocean outpace PP.

However, in direct contrast to this, we find an apparent inverse relationship between PP and export efficiency exists, driven by LP‐HE regimes. HP‐HE regimes, which should be common if the canonical theory that export efficiency increases with PP is correct, only occur in <4% of our data set. Instead, the inverse pattern we find implies that PP increases more rapidly than export flux, that is, PP can become decoupled from the processes acting to remineralize organic carbon in the upper ocean. The scenario of PP outpacing export processes could occur, for example, when phytoplankton growth outpaces zooplankton grazing, such as at the start of a phytoplankton bloom (Franks, 2001; Henson et al., 2015; Ward et al., 2014). If zooplankton growth lags behind phytoplankton growth, a larger proportion of PP may be directly exported rather than grazed, resulting in high export efficiency. This mechanism depends on direct export of phytoplankton cells, either through individual cells sinking or via aggregation of cells, which may not be feasible for small, nonaggregating phytoplankton (Jackson et al., 2015; Laurenceau‐Cornec et al., 2015). Here we find that at the global scale, LP‐HE regimes are associated with relatively high nitrate availability (Figure 2a) and low abundances of macrozooplankton and bacteria relative to lower export efficiency regimes (Figures 3e and 3f), consistent with the concept that LP‐HE conditions occur early in the phytoplankton growing season, before export processes have ramped up. Under these conditions a decoupling between PP and upper ocean remineralization processes can exist, so that although PP is low, much of it is exported.

The size structure of the phytoplankton community, and in particular the dominance of large cells and diatoms, has been implicated as a control on short‐term high export events (e.g., Boyd & Newton, 1995; Buesseler, 1998). In our global scale analysis, although HP‐HE regimes are associated with a higher proportion of microplankton, LP‐HE regimes are not (Figure 3a), and our results therefore suggest that large phytoplankton alone are insufficient to generate a high export event. Instead, low abundances of macrozooplankton and bacteria may be a necessary condition to enable decoupling of PP and export. This mechanism assumes that the abundance of zooplankton is correlated with grazing pressure on phytoplankton, the rate of fecal pellet production and the contribution of fecal pellets to export flux, as demonstrated experimentally (e.g., Cavan et al., 2015; Ebersbach & Trull, 2008; Landry, 1995). In high latitudes, both macrozooplankton and bacteria show seasonal cycles: macrozooplankton overwinter in many high latitude regions, awakening from diapause in spring (e.g., Falk‐Petersen et al., 2009; Johnson & Checkley, 2004), and a seasonal cycle of increasing bacterial abundance and production in spring has been observed (e.g., Garneau et al., 2008; Mevel et al., 2008). Unfortunately, there are insufficient temporally resolved observations in our data set to examine this potential seasonality. LP‐HE regimes may also be associated with transient, nonbiological processes which may contribute to high export efficiency, for example, the mixed layer pump (Dall'Olmo et al., 2016; Giering et al., 2016), in which variability in the mixed layer depth results in detrainment of particles. However, our results point to the driver of LP‐HE regimes as a decoupling of PP from the processes of upper ocean remineralization occurring via macrozooplankton and bacteria.

LP‐LE and HP‐LE regimes tend to occur in specific regions: the equatorial/oligotrophic Atlantic for LP‐LE and equatorial Pacific and Arabian Sea for HP‐LE (Figure 1). Low export regimes are therefore found either in oligotrophic areas which have little seasonality, with low PP year round, or in equatorial regions which have relatively constant high PP. This implies that the biological mediators of remineralization are “in step” with the primary producers and the system is coupled, reducing export. The equatorial Pacific and Arabian Sea can experience both HP‐LE and HP‐ME conditions; the shift into moderate export efficiency regimes coincides with a decline in both bacterial and macrozooplankton abundance, implying that seasonal (de‐)coupling of PP and export can occur in these regions.

In contrast to HP‐LE conditions, in LP‐LE regimes picoplankton make up a higher proportion of PP than in other regimes (Figure 3c), and macrozooplankton and bacteria abundance are high compared to LP‐HE regimes (Figures 3e and 3f). Low productivity conditions are also associated with Si:N drawdown ratio >1 (Figure 2b). This implies that oversilicification of diatoms may be occurring, potentially due to iron limitation (Brzezinski, 1985), which could explain the low PP. Oversilicified diatoms are expected to have increased sinking speed and therefore may be exported more efficiently (Brzezinski et al., 2015), resulting in high export efficiency, although they may contain less carbon than their thin‐shelled counterparts (Assmy et al., 2013). We find however that Si:N > 1 is associated with low export efficiency regimes, implying that coupling between PP and remineralization processes remains strong and export efficiency is correspondingly low.

In our results, a high abundance of macrozooplankton is associated with low export efficiency (Figure 3f), implying intense recycling of organic carbon in the upper ocean, however zooplankton also produce carbon‐rich fecal pellets which have been implicated as a major vector for organic carbon to the mesopelagic (e.g., Belcher et al., 2017; Cavan et al., 2017; Ducklow et al., 2001; Turner, 2015). Two scenarios could explain our apparently contradictory results. Either the fecal pellets produced in low productivity periods or locations are loosely packaged and fragment easily into suspended particles (Belcher et al., 2016; Francois et al., 2002) or diel vertical migration by zooplankton results in excretion of pellets below 100 m (i.e., “active flux”; sensu Steinberg et al., 2000). Although we cannot address either scenario with our data set, we find that low export efficiency is associated with high abundance of macrozooplankton and bacteria, which implies that these organisms are important in controlling export of organic carbon from the upper ocean (see also, e.g., Buesseler & Boyd, 2009; Legendre & Le Fèvre, 1995; Steinberg et al., 2008).

High export efficiency conditions can also occur during high productivity periods (i.e., HP‐HE). Our data suggest that HP‐HE regimes are characterized by high abundances of mesozooplankton (Figures 3d–3f); however, the high export efficiency implies that the recycling pathway is not activated. This suggests possible hypotheses for the existence of HP‐HE conditions: Either large quantities of dense, tightly packaged fecal pellets are produced which sink rapidly out of the upper ocean thus avoiding remineralization, or particulate organic material is passively exported to depth by subduction or advection (Omand et al., 2015; Stukel et al., 2017). The rarity of HP‐HE conditions in our data set (<4%) implies that they are only serendipitously sampled during research cruises and are therefore likely to be transient in nature, which perhaps points to variability in the physical environment as the mechanism.

The Southern Ocean can experience both LP‐HE and low export efficiency regimes, which may offer additional insight into the potential controlling factors. Both LP‐ME/LP‐HE and HP‐ME conditions were encountered (according to our analysis) during a cruise that took place south of Tasmania in summer 2007. During the SAZ‐Sense expedition, Jacquet et al. (2011) report that station P2 was characterized by low PP but relatively high export flux (i.e., high export efficiency, suggesting LP‐ME/LP‐HE conditions) and was dominated by diatoms and nanoplankton but with low zooplankton and very low bacterial abundances. The particle flux at P2 was uniquely (for this cruise) dominated by intact phytoplankton cells and small fecal aggregates, consistent with weak grazing conditions. At a station (P3), 1,100‐km distant from P2, high PP but lower export (HP‐ME) conditions existed under iron replete conditions. Low microplankton and high nanoplankton abundance was encountered, along with the highest bacterial and zooplankton abundances recorded during the cruise. Fecal aggregates dominated the flux at P3, with almost no phytoplankton cells, consistent with tight grazing control of PP (Jacquet et al., 2011). The observed ecosystem structure mirrors our global meta‐analysis, while the observed flux composition supports our interpretation of the role of (de‐)coupling of PP and remineralization processes in driving export efficiency.

Other intensively studied sites yield varying consistency with our results. For example, at the Bermuda Atlantic Time Series station, the export efficiency regime varies between LP‐LE and LP‐ME. Diatom abundance increases and bacterial abundance declines as conditions move toward higher export efficiency, consistent with our results, suggesting that recycling processes via bacterial activity declined, allowing higher export. Moreover, a shift toward larger cells could facilitate faster sinking rates and hence higher export fluxes. However, little change was observed in mesozooplankton biomass, in contrast to our results (Madin et al., 2001; Steinberg et al., 2001). Temporal variability in export efficiency regime is however relatively low at Bermuda Atlantic Time Series. In contrast, at the JGOFS site in the northeast Atlantic, observed conditions shifted from HP‐LE to HP‐HE, corresponding with an increase in mesozooplankton and bacterial abundance (Buesseler et al., 1992; Lochte et al., 1993). While the former observation is consistent with our results, the latter is not (Figure 3). Finally, an intensive month‐long study in the Arctic found a shift from HP‐LE to HP‐HE regimes associated with a decrease in mesozooplankton abundance and an increase in bacteria (Le Moigne et al., 2015), that is, opposite to our meta‐analysis results. These contrasting levels of (dis)agreement with our global scale meta‐analysis further emphasizes our conclusion that differences in conditions associated with export regimes apparent at the global scale are not necessarily reflected at the regional scale.

A previous modelling study examining seasonal changes in the export efficiency found that the early phase of the high latitude bloom was characterized by low export efficiency (Henson et al., 2015). The authors argue that as zooplankton populations are still low early in the year, production is retained in the upper ocean due to a lack of processing by zooplankton. This is in agreement with results from a mesocosm study, which noted that decoupling at the start of the spring bloom resulted in a low export efficiency (Stange et al., 2017). High zooplankton abundance may also drive low export efficiency by facilitating surface recycling of organic carbon or alternatively high export efficiency through production of fecal pellets (Steinberg & Landry, 2017). Here, however, we find that low abundance of macrozooplankton (and bacteria) is associated with high export efficiency, implying that PP is being exported without significant reworking or remineralization, presumably as aggregates (as also suggested by the SAZ‐Sense data set and, for example, Briggs et al., 2011). Our results therefore imply that incorporating upper ocean particle aggregation into biogeochemical models may be fruitful (Jackson & Burd, 2015).

The inverse relationship we find between PP and export efficiency is directly opposite to the positive relationship typically used in satellite data‐based empirical representations of export (e.g., Dunne et al., 2005; Laws et al., 2000). These models do not include LP‐HE regimes which implies that they may underestimate export flux, particularly during periods of decoupling between PP and export, such as early in the phytoplankton growing season. Although LP‐HE regimes are likely transient in nature, these episodic high export efficiency events may contribute a significant proportion of total flux (e.g., Rembauville et al., 2015; Smith et al., 2013) and therefore attempts should be made to incorporate them into empirical and mechanistic models of the biological carbon pump.

At the regional scale, export efficiency generally varied between two states within either the high or low productivity regime (Table S3). In the subpolar biome, the conditions which coincided with shifts in export state generally reflected those found in the global scale analysis, that is, high export efficiency is associated with a large proportion of nitrate remaining at the time of sampling, lower microplankton proportion, and increased mesozooplankton biomass (Figure 4 and Table S2). In the polar biome, LP‐HE conditions are distinguished from LP‐ME by higher bacterial and macrozooplankton abundance. This is in contrast to the global scale analysis, where LP‐HE regimes are found to be characterized by lower bacterial and macrozooplankton abundance (Figures 4 and S10). Ecosystem conditions were not statistically different (at the 95% level) between the two states in the equatorial (HP‐LE/HP‐ME) and oligotrophic (LP‐LE/LP‐ME) biomes (Table S2). This suggests that a factor which we have not included in the analysis may be driving the shift from low to moderate export efficiency in these biomes whilst maintaining the same PP regime (this point is returned to in the next paragraph). Furthermore, the differences in conditions in differing export regimes apparent at the global scale are generally not reflected at the regional scale (with the possible exception of the subpolar biome). This finding implies that region‐specific parameterizations of export efficiency may improve biogeochemical model representation of the biological carbon pump.

We note that our results may be influenced by the uncertainties associated with small sample sizes, particularly at the regional scale. Additionally, our meta‐analysis approach cannot account for all the complexities of the processes thought to influence export flux, such as the interplay between gravitational settling and particle type or size (McDonnell & Buesseler, 2010), details of the community structure (e.g., type of zooplankton, characteristics of different diatom species, and TEP producers; De La Rocha & Passow, 2007; Turner, 2015), transport of organic carbon via the diel vertical migration of zooplankton (Steinberg et al., 2000), export of dissolved organic carbon (Roshan & DeVries, 2017), or the nonbiological transport of particles below the mixed layer by detrainment or subduction (Dall'Olmo et al., 2016; Omand et al., 2015). Finally, our data set consists of discrete data points, biased temporally toward spring and summer at high latitudes, and spatially toward the Atlantic and equatorial Pacific. Data sparsity also compels us to use climatological data sets, thus excluding interannual variability in conditions. In addition, due to the differing availability of in situ observations, some variables were averaged over greater spatial or temporal scales than others, for example, bacterial abundance was averaged over a 3° area centered on the sampling point, rather than the 1° area used for other ecosystem variables. Mesoscale variability, which may be important in driving variability in export efficiency (Buesseler et al., 2008; Maiti et al., 2008), is therefore unaccounted for in our analysis. We also cannot directly test for seasonal scale effects in our data, such as the hypothesis that LP‐HE regimes occur early in the phytoplankton growing season. This data sparsity may have implications for our interpretation of the results. For example, HP‐HE regimes occur mostly in the Atlantic and LP‐HE regimes predominate in the Southern Ocean. This may reflect either a specific and fundamental difference between the Atlantic and Southern oceans in ecosystem interactions and its role in setting export efficiency, or it may arise simply from a sampling bias. LP‐HE and HP‐HE regimes are relatively rare in our data set (of 821 data points, ~10% are LP‐HE and 4% are HP‐HE), implying that they may be transient or episodic in nature. Alternatively, the sampling bias in our data set could be underrepresenting these high export efficiency regimes if they occur commonly but at times and places which are not frequently sampled, for example, high‐latitude winter. Unfortunately, the sparsity of our data set limits our ability to disentangle these possibilities. This points toward the need for seasonal scale, temporally resolved particle flux data, particularly at high latitudes or in dynamic systems. Given the logistical constraints of ship‐board measurements of export flux, the emerging use of autonomous systems, such as Bio‐Argo floats (e.g., Dall'Olmo & Mork, 2014) and gliders (e.g., Alkire et al., 2014), for biogeochemical studies provides an opportunity for investigating the characteristics of seasonal variability in export efficiency and the role of episodic events.

**Figure 5:**
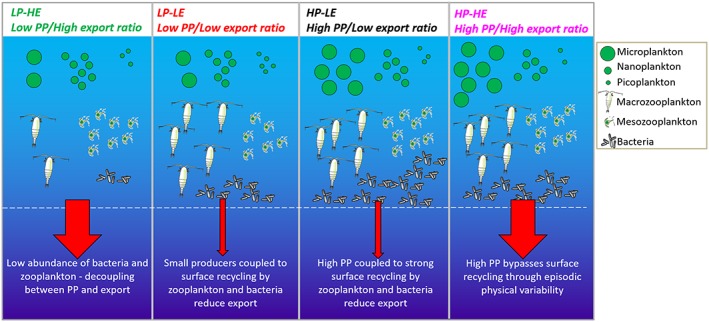
Schematic depicting the four extremes of the global primary productivity‐export efficiency regimes identified in Figure 1b and the corresponding ecological conditions. Moderate export efficiency conditions shown in Figure 1 are considered intermediate cases to the high and low export efficiency extrema.

In summary, we find that the export efficiency can be highly variable depending on the degree of (de‐)coupling between primary producers and upper ocean remineralization by zooplankton and bacteria (see Figure 5 for a schematic summary). Our results confirm that export efficiency is not determined solely by phytoplankton community composition but rather by the whole upper ocean ecosystem structure. Low export efficiency is associated with high abundance of macrozooplankton and bacteria, implying that these organisms play a key role in setting the efficiency of organic carbon export from the upper ocean.

## Supporting information



Supporting Information SI

Data Set S1
