# Supplementary material for: Drivers of Carbon Export Efficiency in the Global Ocean
Source: Global Biogeochem Cycles. 2019 Jul 22;33(7):891–903. doi: 10.1029/2018GB006158 (PMC7006809; doi:10.1029/2018GB006158)
Supplement: Supplementary file 1 — Supporting Information SI [file GBC-33-891-s001.pdf]

Global Biogeochemical Cycles

Supporting Information for

Drivers of carbon export efficiency in the global ocean

Stephanie Henson[1], Fred Le Moigne[2,3], Sarah Giering[1]

[1] National Oceanography Centre, European Way, Southampton, SO14 3ZH, UK

[2] GEOMAR Helmholtz Centre for Ocean Research Kiel, Kiel, Germany

[3] Now at: Mediterranean Institute of Oceanography, UM 110, Aix Marseille Univ., Université de Toulon, CNRS, IRD, 13288, Marseille, France

Contents of this file

Figures S1 to S10
Tables S1 to S3

Introduction

Supplementary figures and tables for 'Drivers of carbon export efficiency in the global ocean'

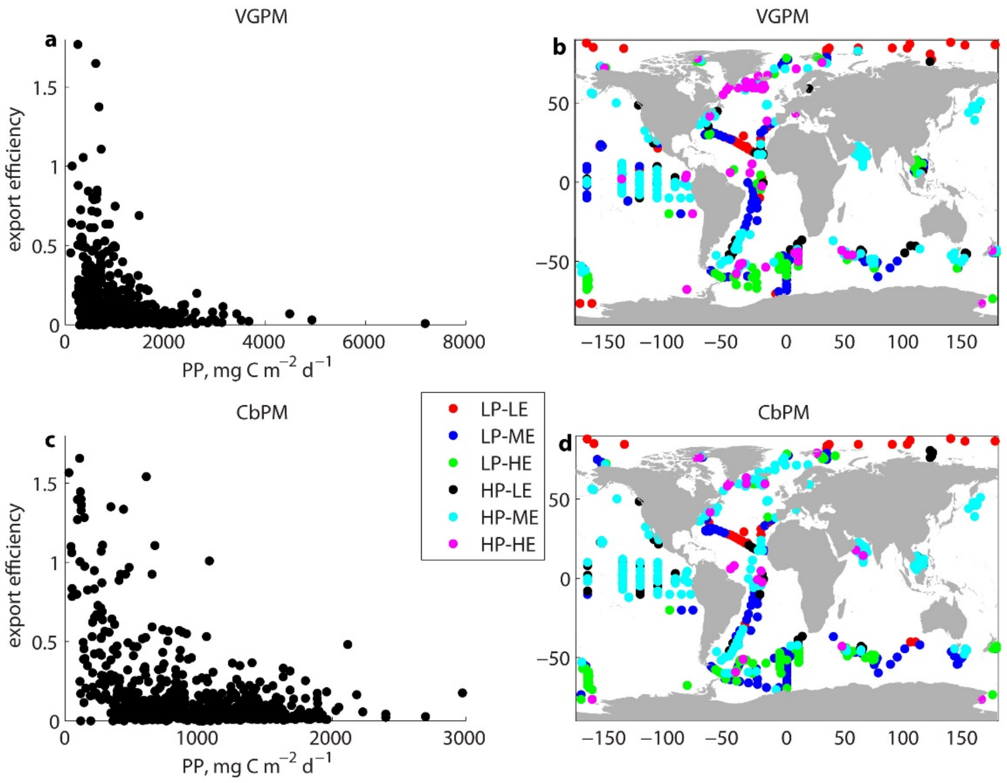

Figure S1: Alternative satellite-derived primary productivity products plotted against export efficiency: a) Using VGPM (Vertically Generalised Production Model; Behrenfeld and Falkowski, 1997) and c) CbPM (Carbon-based Production Model-2; Westberry et al., 2008). Location of *in situ* samples marked in the same colour scheme as for Figure 1 for b) VGPM data and d) CbPM data. See Methods for definition of low/high regimes.

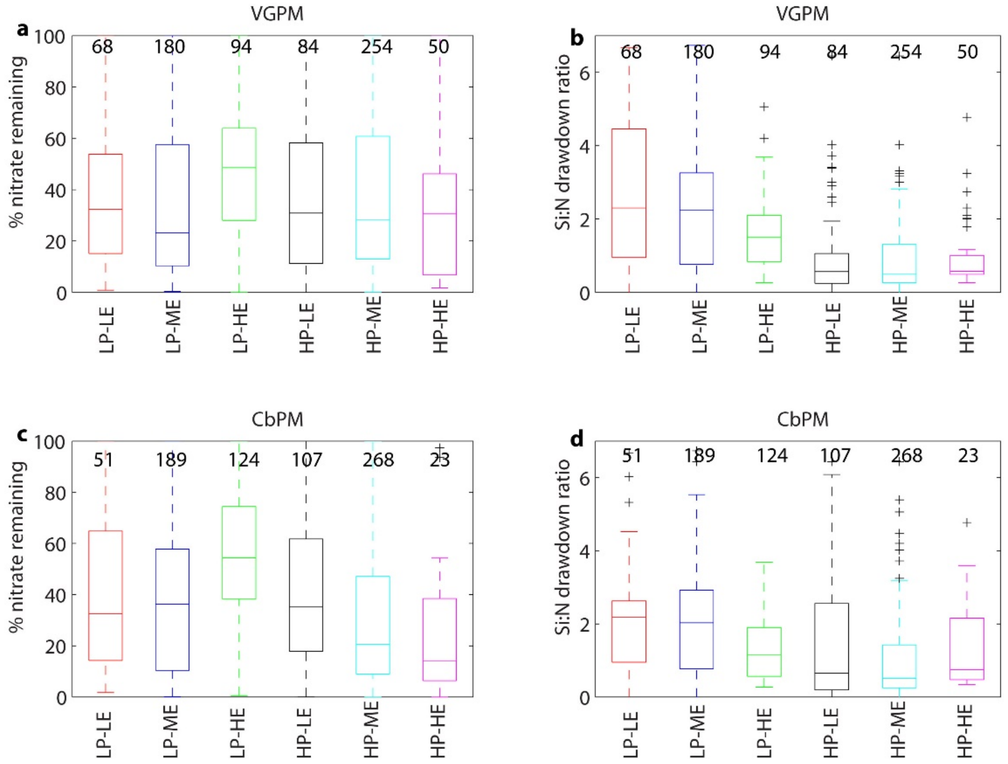

Figure S2: Box plots showing the mean and range in a) and c) percentage of maximum mixed layer nitrate remaining at time of export flux sampling and b) and d) mixed layer silicate:nitrate drawdown ratio for each of the export efficiency regimes for alternative satellite PP estimates (VGPM - Vertically Generalised Production Model; Behrenfeld and Falkowski, 1997, CbPM - Carbon-based Production Model-2; Westberry et al., 2008).

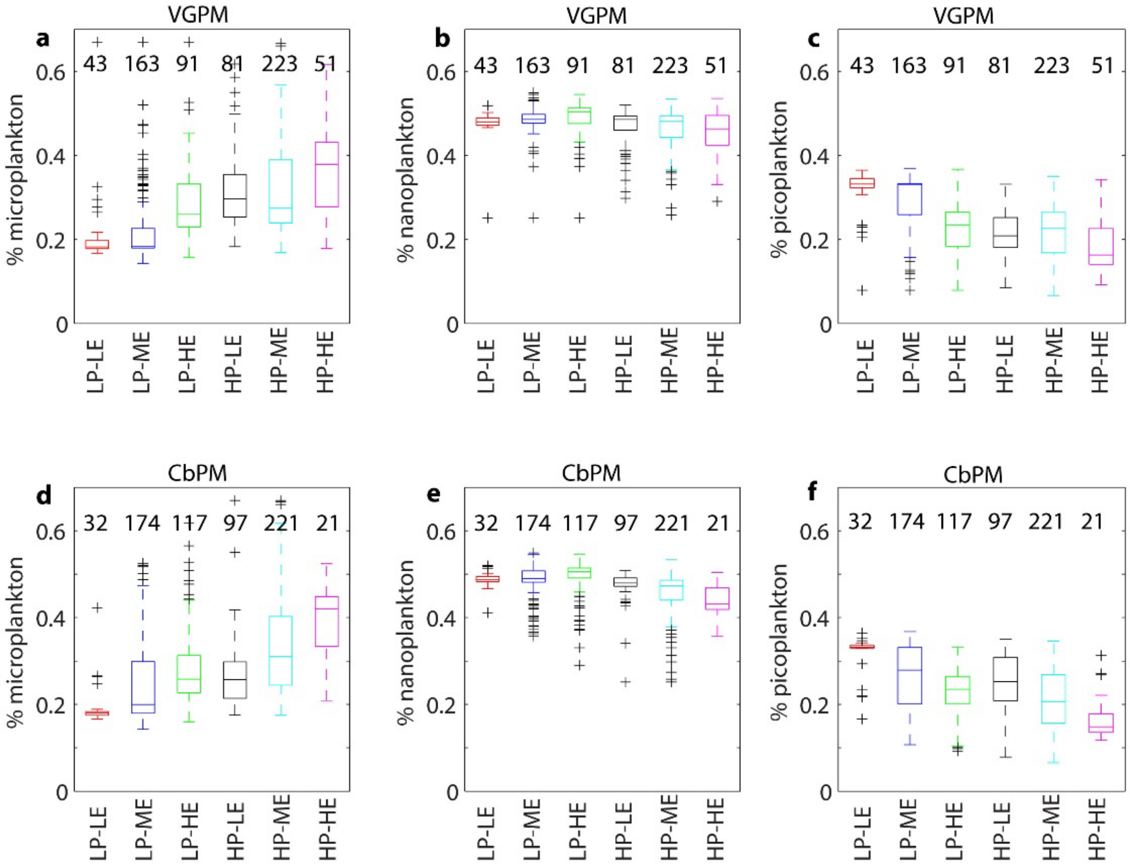

Figure S3: Box plots showing the mean and range in proportion of primary production assigned to 3 phytoplankton size classes – a) and d) microplankton; b) and e) nanoplankton; c) and f) picoplankton – for alternative satellite PP estimates (VGPM - Vertically Generalised Production Model, Behrenfeld and Falkowski, 1997; CbPM - Carbon-based Production Model-2, Westberry et al., 2008) in each of the export efficiency regimes. Phytoplankton size estimates are taken from satellite-derived data (Uitz et al., 2010).

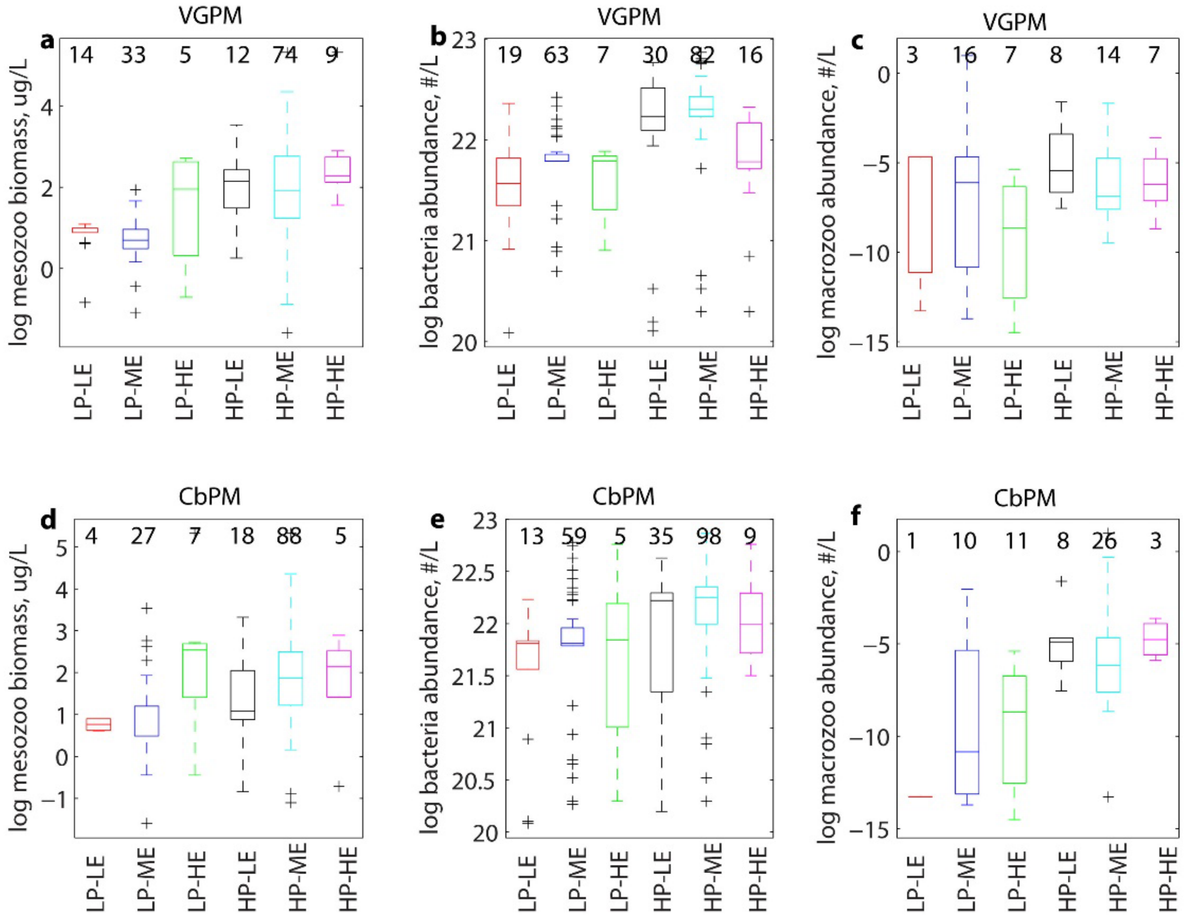

Figure S4: Box plots showing the mean and range in a) and d) mesozooplankton biomass; b) and e) bacterial abundance; c) and f) macrozooplankton abundance for alternative satellite PP estimates (VGPM - Vertically Generalised Production Model, Behrenfeld and Falkowski, 1997; CbPM - Carbon-based Production Model-2, Westberry et al., 2008) in each of the export efficiency regimes. Zooplankton and bacteria data are taken from Buitenhuis et al. (2012), Moriarty et al. (2013) and Moriarty and O'Brien (2013).

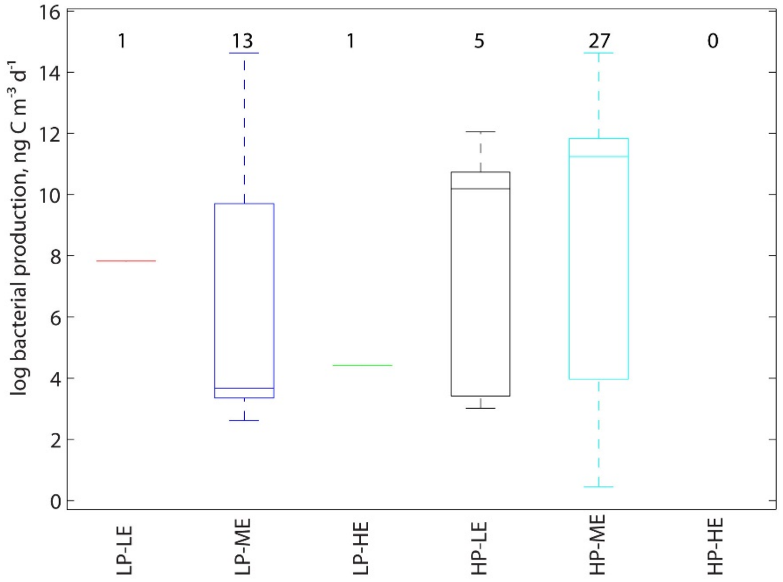

Figure S5: Box plots showing the mean and range in bacterial production in each export efficiency regime. Numbers at the top of the plot indicate how many data points occur in each category.

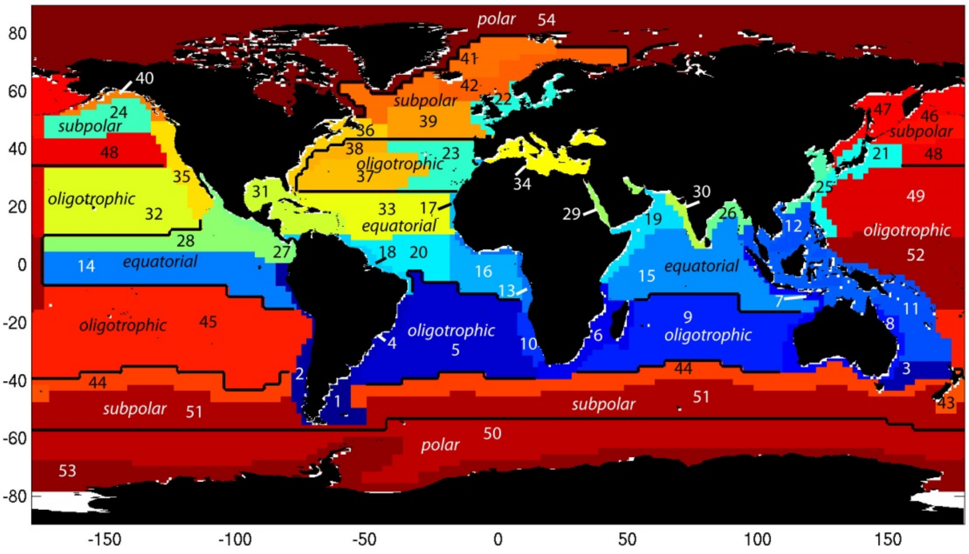

Figure S6: Map showing the biomes into which Longhurst's provinces (Longhurst, 2007) were aggregated. The Longhurst provinces assigned to each biome are: Equatorial – 14, 15, 16, 17, 18, 19, 20, 28, 30, 33; Oligotrophic – 5, 9, 23, 32, 37, 38, 45, 49, 52; Subpolar – 24, 36, 39, 41, 42, 43, 44, 46, 47, 48, 51; Polar – 50, 53, 54. Provinces not listed as assigned to a biome are coastal regions and are excluded from the analysis.

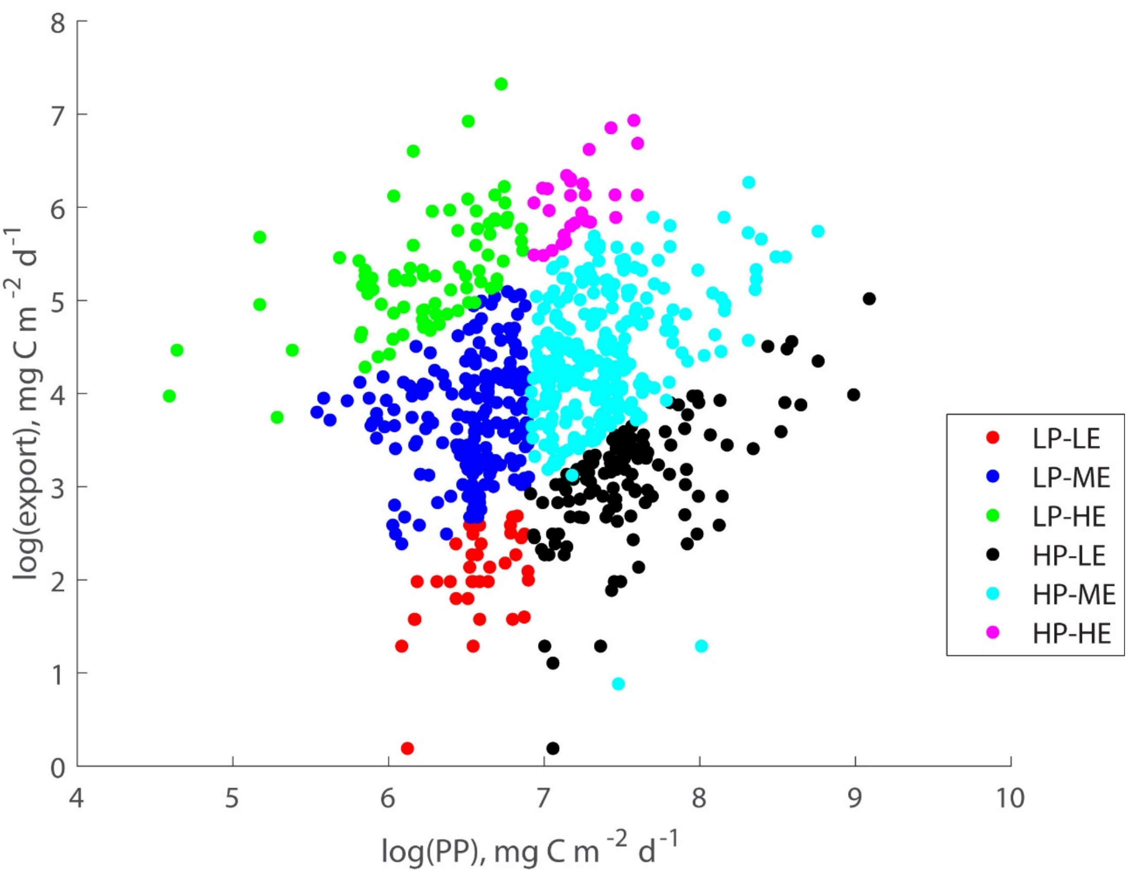

Figure S7: Log-log plot (natural log) of PP and export flux coloured by export efficiency regime.

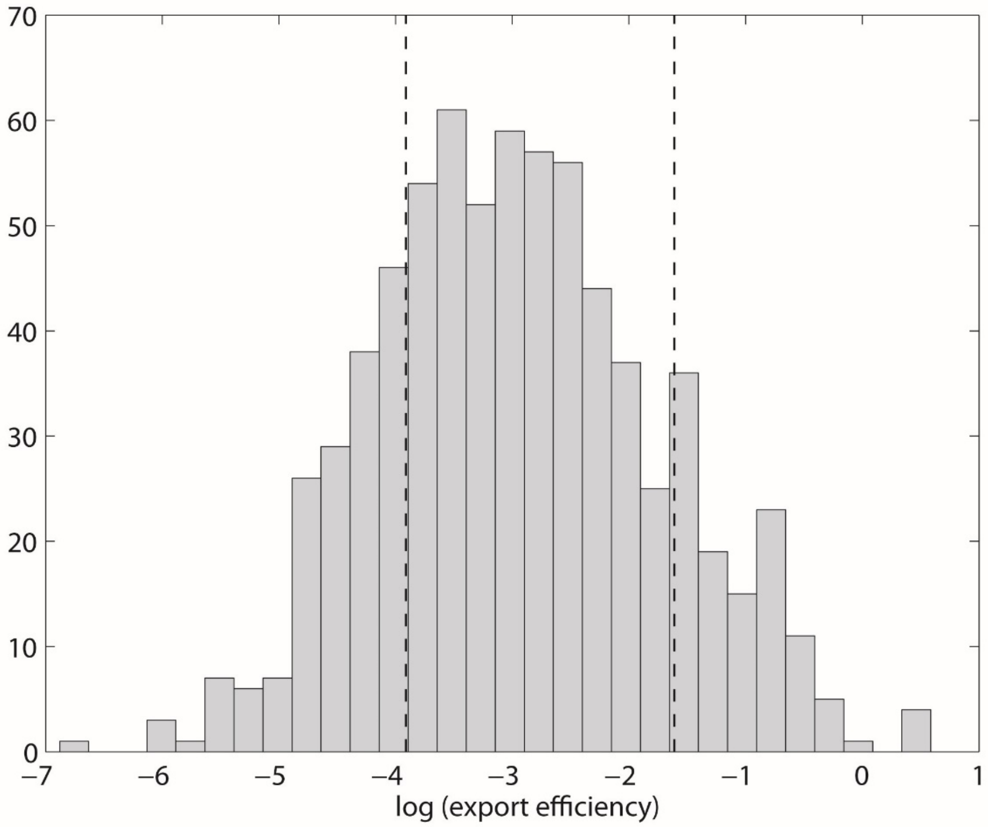

Figure S8: Histogram of natural log of export efficiency measurements (data are log normally distributed). Dashed lines show the low and high export efficiency thresholds (see Methods section for more details).

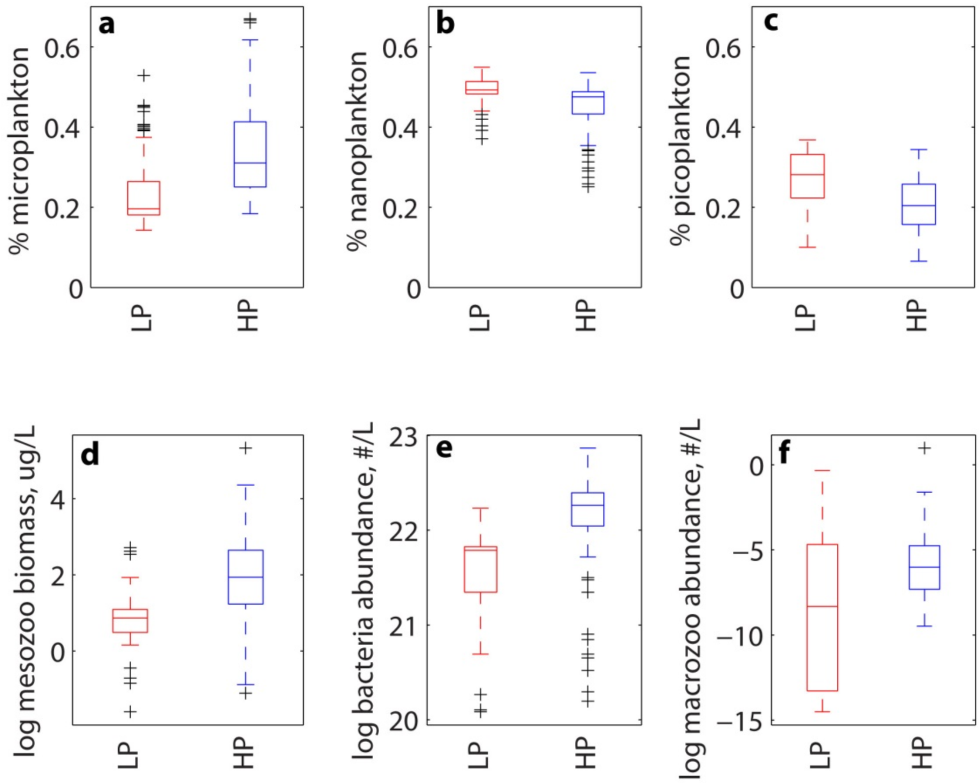

Figure S9: Box plots showing the mean and range in proportion of primary production assigned to 3 phytoplankton size classes: a) microplankton; b) nanoplankton; c) picoplankton, d) mesozooplankton biomass, e) bacterial abundance and f) macrozooplankton abundance in low and high productivity groups (LP and HP, respectively). ANOVA tests demonstrated that the differences in the means between the groups are statistically significant at the 95% level for all variables.

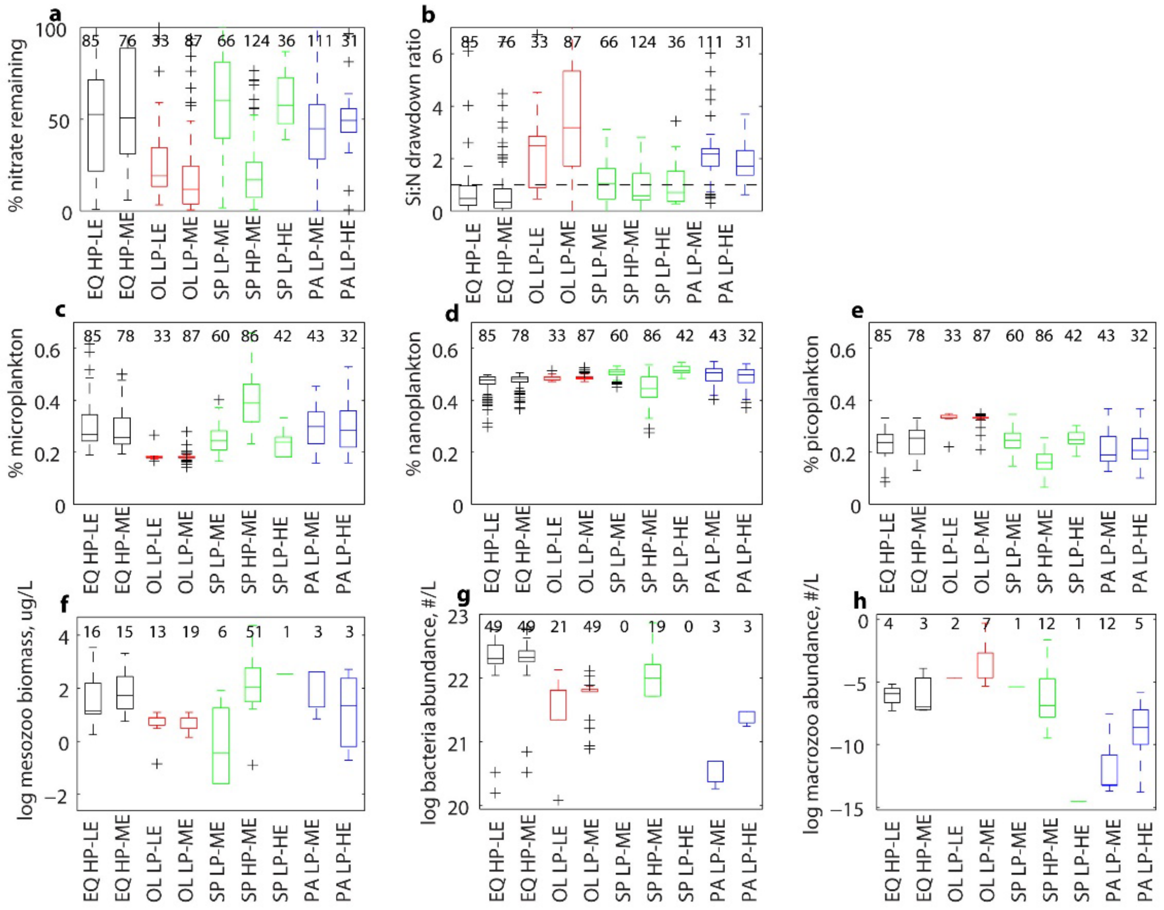

Figure S10: Box plots showing the mean and range in a) percentage of maximum mixed layer nitrate remaining at time of export flux sampling; b) mixed layer silicate:nitrate drawdown ratio; proportion of primary production assigned to 3 phytoplankton size classes – c) micro, d) nano and e) picoplankton – taken from satellite-derived estimates (Uitz et al., 2010); f) mesozooplankton biomass, g) bacterial abundance and h) macrozooplankton abundance (Buitenhuis et al., 2012; Moriarty et al., 2013; Moriarty and O'Brien, 2013). Biome codes are: EQ – equatorial, OL – oligotrophic, SP – subpolar and PA – polar. Statistical significance of the difference in means between regimes was tested using ANOVA; results are shown in Table S2.

%nit	LP-LE	LP-ME	LP-HE	HP-LE	HP-ME	HP-HE
LP-LE	-	0.12	**<0.01**	0.18	0.94	*0.07*
LP-ME	0.12	-	**<0.01**	0.95	**<0.01**	**<0.01**
LP-HE	**<0.01**	**<0.01**	-	**<0.01**	**<0.01**	**<0.01**
HP-LE	0.18	0.95	**<0.01**	-	**0.04**	**<0.01**
HP-ME	0.94	**<0.01**	**<0.01**	**0.04**	-	**0.04**
HP-HE	*0.07*	**<0.01**	**<0.01**	**<0.01**	**0.04**	-

Micro	LP-LE	LP-ME	LP-HE	HP-LE	HP-ME	HP-HE
LP-LE	-	**<0.01**	**<0.01**	**<0.01**	**<0.01**	**<0.01**
LP-ME	**<0.01**	-	**<0.01**	**<0.01**	**<0.01**	**<0.01**
LP-HE	**<0.01**	**<0.01**	-	**<0.01**	**<0.01**	**<0.01**
HP-LE	**<0.01**	**<0.01**	**<0.01**	-	**0.03**	**0.01**
HP-ME	**<0.01**	**<0.01**	**<0.01**	**0.03**	-	0.13
HP-HE	**<0.01**	**<0.01**	**<0.01**	**0.01**	0.13	-

Meso	LP-LE	LP-ME	LP-HE	HP-LE	HP-ME	HP-HE
LP-LE	-	0.93	0.12	**<0.01**	**<0.01**	**<0.01**
LP-ME	0.93	-	0.15	**<0.01**	**<0.01**	**<0.01**
LP-HE	0.12	0.15	-	0.79	0.29	0.43
HP-LE	**<0.01**	**<0.01**	0.79	-	**0.05**	0.20
HP-ME	**<0.01**	**<0.01**	0.29	**0.05**	-	0.99
HP-HE	**<0.01**	**<0.01**	0.43	0.20	0.99	-

S:N	LP-LE	LP-ME	LP-HE	HP-LE	HP-ME	HP-HE
LP-LE	-	**0.03**	0.79	0.22	0.18	0.77
LP-ME	**0.03**	-	**<0.01**	**<0.01**	**<0.01**	**0.02**
LP-HE	0.79	**<0.01**	-	**0.04**	**0.02**	0.20
HP-LE	0.22	**<0.01**	**0.04**	-	0.68	0.36
HP-ME	0.18	**<0.01**	**0.02**	0.68	-	0.35
HP-HE	0.77	**0.02**	0.20	0.36	0.35	-

Nano	LP-LE	LP-ME	LP-HE	HP-LE	HP-ME	HP-HE
LP-LE	-	**0.03**	**0.01**	**<0.01**	**<0.01**	**<0.01**
LP-ME	**0.03**	-	**0.04**	**<0.01**	**<0.01**	**<0.01**
LP-HE	**0.01**	**0.04**	-	**<0.01**	**<0.01**	**<0.01**
HP-LE	**<0.01**	**<0.01**	**<0.01**	-	0.39	0.27
HP-ME	**<0.01**	**<0.01**	**<0.01**	0.39	-	0.51
HP-HE	**<0.01**	**<0.01**	**<0.01**	0.27	0.51	-

Bact	LP-LE	LP-ME	LP-HE	HP-LE	HP-ME	HP-HE
LP-LE	-	0.16	0.74	**<0.01**	**<0.01**	0.17
LP-ME	0.16	-	0.25	**<0.01**	**<0.01**	0.41
LP-HE	0.74	0.25	-	**0.01**	*0.07*	0.13
HP-LE	**<0.01**	**<0.01**	**0.01**	-	*0.09*	**0.01**
HP-ME	**<0.01**	**<0.01**	*0.07*	*0.09*	-	0.13
HP-HE	0.17	0.41	0.13	**0.01**	0.13	-

Pico	LP-LE	LP-ME	LP-HE	HP-LE	HP-ME	HP-HE
LP-LE	-	**<0.01**	**<0.01**	**<0.01**	**<0.01**	**<0.01**
LP-ME	**<0.01**	-	**<0.01**	**<0.01**	**<0.01**	**<0.01**
LP-HE	**<0.01**	**<0.01**	-	0.16	**<0.01**	**<0.01**
HP-LE	**<0.01**	**<0.01**	0.16	-	**<0.01**	**<0.01**
HP-ME	**<0.01**	**<0.01**	**<0.01**	**<0.01**	-	**0.04**
HP-HE	**<0.01**	**<0.01**	**<0.01**	**<0.01**	**0.04**	-

Macro	LP-LE	LP-ME	LP-HE	HP-LE	HP-ME	HP-HE
LP-LE	-	0.22	*0.09*	0.70	0.58	0.29
LP-ME	0.22	-	0.57	**0.03**	**0.01**	0.42
LP-HE	*0.09*	0.57	-	**<0.01**	**<0.01**	0.18
HP-LE	0.70	**0.03**	**<0.01**	-	0.59	0.30
HP-ME	0.58	**0.01**	**<0.01**	0.59	-	0.60
HP-HE	0.29	0.42	0.18	0.30	0.60	-

Table S1: Table showing results of ANOVA tests on the means of the environmental variables in each export efficiency regime against all other regimes. A multiple comparison procedure was used to reduce the chance of false positives. Variable names are: % nitrate remaining (%nit), Si:N drawdown ratio (Si:N), proportion of PP assigned to microplankton (Micro), proportion of PP assigned to nanoplankton (Nano), proportion of PP assigned to pickoplankton (Pico), mesozooplankton biomass (Meso), bacterial abundance (Bact) and macrozooplankton abundance (Macro). Bold numbers indicate the difference in the mean of the 2 groups are significant at the 95% level; italics indicate they are significant at the 90% level.

	HP-LE:HP-ME	LP-LE:LP-ME	LP-ME:HP-ME	LP-ME:LP-HE	HP-ME:LP-HE	LP-ME:LP-HE
%nit	0.16	*0.08*	**<0.01**	1.00	**<0.01**	0.35
Si:N	0.71	**<0.01**	*0.02*	0.59	0.25	0.53
Micro	0.37	0.19	**<0.01**	*0.08*	**<0.01**	0.85
Nano	0.38	0.47	**<0.01**	**0.02**	**<0.01**	0.28
Pico	0.43	0.49	**<0.01**	0.23	**<0.01**	0.66
Meso	0.35	0.89	**<0.01**	0.15	0.75	0.48
Bact	0.64	0.21	n/a	0.15	n/a	**0.01**
Macro	0.98	0.53	0.72	n/a	**<0.01**	**0.04**

Table S2: Table showing results of ANOVA tests on the means of environmental variables for the dominant export efficiency states in each biome. Variable names are: % nitrate remaining (%nit), Si:N drawdown ratio (Si:N), proportion of PP assigned to microplankton (Micro), proportion of PP assigned to nanoplankton (Nano), proportion of PP assigned to pickoplankton (Pico), mesozooplankton biomass (Meso), bacterial abundance (Bact) and macrozooplankton abundance (Macro). Bold numbers indicate the difference in the mean of the 2 groups are significant at the 95% level; italics indicate they are significant at the 90% level; n/a indicates that insufficient data exist to perform an ANOVA test.

	LP-LE	LP-ME	LP-HE	HP-LE	HP-ME	HP-HE
**Equatorial**	7	5	0	85	78	6
**Oligotrophic**	33	86	4	10	11	1
**Subpolar**	5	70	42	12	127	10
**Polar**	0	113	33	3	13	12

Table S3: Number of data points in each biome that fall into each export efficiency regime category. In each biome, two export efficiency states dominate, which are highlighted in bold.
